# Transition rate theory, spectral analysis, and reactive paths

**DOI:** 10.1063/5.0084209

**Published:** 2022-04-04

**Authors:** Benoît Roux

**Affiliations:** Department of Biochemistry and Molecular Biology, Department of Chemistry, The University of Chicago, 5735 S Ellis Ave., Chicago, Illinois 60637, USA

## Abstract

The kinetics of a dynamical system dominated by two metastable states is examined from the perspective of the activated-dynamics reactive flux formalism, Markov state eigenvalue spectral decomposition, and committor-based transition path theory. Analysis shows that the different theoretical formulations are consistent, clarifying the significance of the inherent microscopic lag-times that are implicated, and that the most meaningful one-dimensional reaction coordinate in the region of the transition state is along the gradient of the committor in the multidimensional subspace of collective variables. It is shown that the familiar reactive flux activated dynamics formalism provides an effective route to calculate the transition rate in the case of a narrow sharp barrier but much less so in the case of a broad flat barrier. In this case, the standard reactive flux correlation function decays very slowly to the plateau value that corresponds to the transmission coefficient. Treating the committor function as a reaction coordinate does not alleviate all issues caused by the slow relaxation of the reactive flux correlation function. A more efficient activated dynamics simulation algorithm may be achieved from a modified reactive flux weighted by the committor. Simulation results on simple systems are used to illustrate the various conceptual points.

## I. INTRODUCTION

The dynamics of a complex system with two long-lived metastable states is a classical problem that is often represented phenomenologically by the kinetic model,
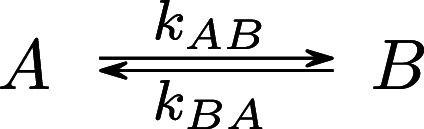
with the overall relaxation time $\tau^{*}={\left(k_{AB}+k_{BA}\right)}^{-1}$ and equilibrium probabilities *p*_*A*_ = *k*_*BA*_/(*k*_*AB*_ + *k*_*BA*_) and *p*_*B*_ = *k*_*AB*_/(*k*_*AB*_ + *k*_*BA*_). One of the most important theoretical frameworks to tackle such a problem in complex systems has been Chandler’s activated-dynamics reactive flux formalism.^1^ Assuming that *x* is a good “reaction coordinate” for the system of interest, the forward transition rate *k*_*AB*_ is then expressed as

$$k_{AB}=\kappa \;k_{AB}^{\text{TST}},$$ (1)

where $k_{AB}^{\text{TST}}$ is the transition state theory (TST) rate evaluated at the transition state *x*^†^,^2–4^ and the prefactor *κ* is the transmission coefficient serving as a correction (*κ* ≤ 1). In the context of this analysis, *κ* can be deduced from a time-correlation function evaluated at the molecular time scale, *τ*_m_, expected to be much shorter than the overall relaxation time of the system τ*. For this reason, one of the most attractive features of the activated-dynamics algorithm is that *κ* can be determined from the fate of relatively short trajectories of length *τ*_m_ initiated at the transition state.^1,5,6^

Generally, to characterize the dynamics of complex systems with two long-lived metastable states, one may adopt two different fundamental perspectives. One may choose to focus on the global relaxation time τ* of the system or, alternatively, on the net unidirectional reactive flux *J*_*AB*_ from the state *A* to the state *B*. For a simple two-state kinetic model, the global relaxation time τ* incorporates both the influence of forward *A* → *B* and backward *B* → *A* transitions, reflecting the natural back and forth dynamics occurring spontaneously in the system under equilibrium conditions. In contrast, the forward transition rate *k*_*AB*_, like the mean first passage time (MFPT),^7^ is more directly associated with a specific unidirectional *A* → *B* reactive flux, picturing the kinetic of the system in terms of a “reactant” to a “product” state. In some sense, the first picture seems to simply reflect the system’s natural kinetics, whereas the second picture frames the issue more deliberately by ascribing a directionality to the system’s kinetics.^7^ Of course, both pictures are equivalent because under equilibrium conditions, the forward and backward net unidirectional reactive flux must be equal, *J*_*BA*_ = *J*_*AB*_, and detailed balance implies that *p*_*A*_ *k*_*AB*_ = *p*_*B*_ *k*_*BA*_.

Although this discussion is organized around an exceedingly simple two-state system, it helps illustrate the two different computational and theoretical framework that can be used to characterize the kinetics of highly complex systems. One may choose to attack the problem from the point of view of the global timescales within the system by carrying out a spectral decomposition analysis of the dynamical propagator,^8–12^ or one may consider the net forward flux in one specific direction by deliberately identify boundary reactant and product states and determining the steady-state flux of reactive trajectories between them.^7,13–15^ This construct, which aims its attention toward the set of *A* → *B* reactive trajectories forming the transition path ensemble, is a foundational element of the transition path sampling (TPS) algorithm^16–20^ and transition path theory (TPT).^14^ While the spectral analysis is untainted by the somewhat subjective choice of the boundary states, these theoretical frameworks more directly focus on the specific transition that is the object of interest. This is advantageous in highly complex multistate systems for which a spectral analysis can become obscured by slow processes that are not relevant to the transitions of interest between *A* and *B*. The analysis of the conditions establishing a net steady-state flux between two metastable states leads to the observation that the principal lines of reactive probability current from the state *A* to the state *B* are largely determined by the equilibrium probability and the variations in the committor probability.^7,13–15^ Originally introduced by Onsager in 1938,^21^ and subsequently re-discovered in the late 1990s,^22–24^ the committor probability is a fundamental building block of TPT.^14^ This perspective is a critical insight in the formulation of the string method,^12,25–29^ aimed at determining the “reaction tube” that supports most of the reactive flux from *A* to *B*. It is the objective of the present analysis to shed new light on the characterization the kinetics of complex systems by examining the relationship between the reactive flux formalism, the spectral analysis of Markov models, and the TPT framework.

## II. THEORETICAL DEVELOPMENTS

### A. Reactive flux formalism

For the sake of clarity, it is worth recalling the main elements of the reactive flux formalism for a system with two metastable states *A* and *B*. It is assumed that there is a single “reaction channel” between the two states. Having defined a population operator or indicator function *H*_*B*_ that is equal to 1 when the system is in state *B* and equal to zero when the system is in state *A*, Chandler started by considering the normalized population time-correlation function,^1^

$$C\left(t\right)=\frac{〈\delta H_{B}\left(0\right)\;\delta H_{B}\left(t\right)〉}{〈\delta H_{B}\left(0\right)\;\delta H_{B}\left(0\right)〉},$$ (2)

where *δH*_*B*_(*t*) = *H*_*B*_(*t*) − ⟨*H*_*B*_⟩ is the deviation from the average. Additional useful relations include ⟨*H*_*B*_⟩ = *p*_*B*_, ⟨*H*_*A*_⟩ = ⟨1 − *H*_*B*_⟩ = *p*_*A*_, and ⟨*δH*_*B*_ *δH*_*B*_⟩ = *p*_*A*_ *p*_*B*_. Noting that $C\left(t\right)∼e^{-t/\tau^{*}}$ at long time according to the phenomenology, Chandler argued that the time derivative $\dot{C}\left(t\right)$ must decay from an initial value to a plateau equal to −1/τ* in the limit of *t* → *τ*_m_. In the context of this analysis, *τ*_m_ represents a molecular time scale that must be much shorter than the overall relaxation time of the system $\tau^{*}={\left(k_{AB}+k_{BA}\right)}^{-1}$. Traditionally, one must then define a “reaction coordinate,” *x*(**z**), as a function of a set of collective variables **z**. Once the transition state *x*^†^ is identified, the indicator function *H*_*B*_ can be constructed as

$$H_{B}\left(\text{z}\right)=\theta \left(x\left(\text{z}\right)-x^{†}\right),$$ (3)

where *θ*() is a Heaviside step-function. Assuming that *x* is a good reaction coordinate for the system of interest, the forward transition rate *k*_*AB*_ = *p*_*B*_/τ* is then expressed as

$$\begin{array}{ll} k_{AB} & =\underset{t\to \tau_{\text{m}}}{\lim}\;\frac{1}{p_{A}}\;\left\langle \delta \left(x\left(0\right)-x^{†}\right)\;\dot{x}\left(0\right)\;H_{B}\left(t\right)\right\rangle \\ & =\frac{\rho_{\text{eq}}\left(x^{†}\right)}{\int_{A}\rho_{\text{eq}}\left(x\right)\;dx}\;{\left\langle \dot{x}\;\theta \left(\dot{x}\right)\right\rangle }_{\left(x^{†}\right)}\underset{t\to \tau_{\text{m}}}{\lim}\;\frac{\left\langle \delta \left(x-x^{†}\right)\;\dot{x}\;H_{B}\left(t\right)\right\rangle }{\left\langle \delta \left(x-x^{†}\right)\;\dot{x}\;\theta \left(\dot{x}\right)\right\rangle }\\ & =k_{AB}^{\text{TST}}\;\;\kappa , \end{array}$$ (4)

where *x*^†^ is the position of the transition state, *ρ*_eq_(*x*) is the marginal equilibrium distribution along *x*, $k_{AB}^{\text{TST}}$ is the transition state theory (TST) rate, and *κ* is the transmission coefficient serving as a correction to the transition state theory rate,

$$\kappa =\underset{t\to \tau_{\text{m}}}{\lim}\;\frac{\left\langle \delta \left(x-x^{†}\right)\;\dot{x}\;H_{B}\left(t\right)\right\rangle }{\left\langle \delta \left(x-x^{†}\right)\;\dot{x}\;\theta \left(\dot{x}\right)\right\rangle }.$$ (5)

Alternatively, the *k*_*AB*_ transition rate can also be formulated as the time derivative of the conditional probability that the system will be found in state *B* at time *t*, assuming it was initially in state *A* at time *t* = 0,^30^

$$k_{AB}=\underset{t\to \tau_{\text{m}}}{\lim}\;\frac{d}{dt}\;{\left\langle H_{B}\left(t\right)\right\rangle }_{\left(\text{in }A\text{ at }t=0\right)}$$ (6)

$$\begin{array}{ccc} & = & \underset{t\to \tau_{\text{m}}}{\lim}\;\frac{\left\langle H_{A}\left(0\right){\dot{H}}_{B}\left(t\right)\right\rangle }{\left\langle H_{A}\left(0\right)\right\rangle }\\ & = & \underset{t\to \tau_{\text{m}}}{\lim}\;\frac{1}{p_{A}}\;\left\langle \left(1-H_{B}\left(0\right)\right){\dot{H}}_{B}\left(t\right)\right\rangle \\ & = & \underset{t\to \tau_{\text{m}}}{\lim}\;-\frac{1}{p_{A}}\;\left\langle H_{B}\left(0\right){\dot{H}}_{B}\left(t\right)\right\rangle , \end{array}$$ (7)

where $\left\langle {\dot{H}}_{B}\left(t\right)\right\rangle =0$ was used.

One of the most attractive features of the activated-dynamics algorithm based on Eq. (4) is that *κ* can be determined from the fate of relatively short trajectories of length *τ*_m_ initiated at the transition state *x*^†^.^1,5,6^ On the other hand, the approach may be encountering considerable challenges when the time *τ*_m_ needed to reach the plateau of the time-correlation function of Eq. (5) is too long. While the formalism provides a sound description of the relaxation of a two-state system from a fundamental point of view, the relative measure of success or failure of the approach depends on the rate of convergence of Eq. (5). Methodologies that can help reduce *τ*_m_ and can lead to significantly better statistical properties of the transmission coefficient and the reactive flux estimates are important.^31,32^

### B. Effective propagator and spectral decomposition

The probability density of the system at time *t* is expressed as *ρ*(**x**, **v**; *t*), where **x** and **v** represent the set of coordinates *x*_*i*_ and velocities *v*_*i*_, respectively.^12^ Using **u** ≡ (**x**, **v**) to represents a point in phase space, the forward propagation step (**u** → **u**′) for the probability density from the time *t* to the time *t* + Δ*t* is

$$\rho \left({\text{u}}^{\prime };t+\Delta t\right)=\int d\text{u}\;P_{\Delta t}\left({\text{u}}^{\prime }|\text{u}\right)\;\rho \left(\text{u};t\right).$$ (8)

The elementary propagator for a null time step, *P*_0_(**u**′|**u**), is the identity *δ*(**u**′ − **u**). The dynamical propagation, which we may formally represent as ***ρ***(*t* + Δ*t*) = ***P***_Δ*t*_ · ***ρ***(*t*), obeys the Chapman–Kolmogorov equation for arbitrary times. The propagator with the microscopic time step may be repeatedly applied an arbitrary number of times as $\rho \left(t+n\Delta t\right)={\left(P_{\Delta t}\right)}^{n}\cdot \rho \left(t\right)$. The forward–backward microscopic detailed balance relation, $P_{\Delta t}\left({\text{u}}^{\prime }|\text{u}\right)\;\rho_{\text{eq}}\left(\text{u}\right)=P_{\Delta t}^{†}\left(\text{u}|{\text{u}}^{\prime }\right)\;\rho_{\text{eq}}\left({\text{u}}^{\prime }\right)$, is satisfied, where $P_{\Delta t}^{†}$ is the backward propagator. While a formulation based on the microscopic propagator *P*_Δ*t*_(**u**′|**u**) offers the most complete representation of the reactive paths, it is generally necessary to consider the dynamics projected onto a subspace of reduced dimensionality. We define the effective propagator $P_{\tau }$ for the finite lag-time *τ* within the subspace spanned by a subset of collective variables (CVs) as

$$\begin{array}{ll} P_{\tau }\left({\text{z}}^{\prime }|\text{z}\right) & =\frac{1}{\rho_{\text{eq}}\left(\text{z}\right)}\int d\text{u}\\ & \;\times \int d{\text{u}}^{\prime }\;\delta \left(\tilde{\text{z}}\left({\text{x}}^{\prime }\right)-{\text{z}}^{\prime }\right)P_{\tau }\left({\text{u}}^{\prime }|\text{u}\right)\;\rho_{\text{eq}}\left(\text{u}\right)\;\delta \left(\tilde{\text{z}}\left(\text{x}\right)-\text{z}\right), \end{array}$$ (9)

where $\tilde{\text{z}}\left(\text{x}\right)=\left({\tilde{z}}_{1}\left(\text{x}\right),\ldots ,{\tilde{z}}_{N}\left(\text{x}\right)\right)$ is a vector-valued function that maps every microscopic configuration **x** of the system on a set of values $\tilde{\text{z}}\left(\text{x}\right)$. The reduced probability density of the system at time *t* is expressed as *ρ*(**z**; *t*). The forward propagation step (**z** → **z**′) for the reduced probability density from the time *t* to the time *t* + *τ* is

$$\rho \left({\text{z}}^{\prime };t+\tau \right)=\int d\text{z}\;\;P_{\tau }\left({\text{z}}^{\prime }|\text{z}\right)\;\;\rho \left(\text{z};t\right).$$ (10)

It is assumed that the dynamics within the reduced subspace of the CVs is Markovian with a finite lag-time *τ* and that the propagator obeys the Chapman–Kolmogorov equation, $\rho \left(t+n\tau \right)={\left(P_{\tau }\right)}^{n}\cdot \rho \left(t\right)$. It is assumed that the system is in equilibrium and that we have microscopic detailed balance, $P_{\tau }\left({\text{z}}^{\prime }|\text{z}\right)\;\rho_{\text{eq}}\left(\text{z}\right)=P_{\tau }\left(\text{z}|{\text{z}}^{\prime }\right)\;\rho_{\text{eq}}\left({\text{z}}^{\prime }\right)$, where $\rho_{\text{eq}}\left(\text{z}\right)=\int d\text{x}\;d\text{v}\;\delta \left(\tilde{\text{z}}\left(\text{x}\right)-\text{z}\right)\;\rho_{\text{eq}}\left(\text{x},\text{v}\right)$ is the equilibrium probability in the subspace of the CVs. Under these conditions, the effective propagator $P_{\tau }\left({\text{z}}^{\prime }|\text{z}\right)$ yields a self-consistent representation of the dynamics of the system within this subspace (closure of the dynamical propagation), built on a trajectory generated via the elementary propagator *P*_Δ*t*_(**u**′|**u**) with a time step Δ*t* shorter than *τ*.

In practice, one should seek to determine the smallest possible lag-time *τ* that achieves Markovity for the effective propagator. An important framework to examine this issue is to rely on a spectral decomposition of the effective dynamical propagator.^8,9,12^ The right-eigenvector $\psi_{k}^{\text{R}}\left(\text{z}\right)$ of the operator is defined as^8,9^

$$\lambda_{k}\left(\tau \right)\;\psi_{k}^{\text{R}}\left({\text{z}}^{\prime }\right)=\int d\text{z}\;P_{\tau }\left({\text{z}}^{\prime }|\text{z}\right)\;\psi_{k}^{\text{R}}\left(\text{z}\right),$$ (11)

where the eigenvalue $\lambda_{k}\left(\tau \right)=e^{-\mu_{k}\tau }$. The constants *μ*_*k*_ ≥ 0 represents the associated *τ*-independent intrinsic decay rate of the *n*th eigenmode. The eigenvector $\psi_{1}^{\text{R}}\left(\text{z}\right)$ with the eigenvalue *λ*_1_ = 1 (*μ*_0_ = 0) corresponds to the invariant equilibrium vector, *ρ*_eq_(**z**). The eigenvalues are ordered from the slowest to the fastest process, i.e., 1 = *λ*_1_ > *λ*_2_ > *λ*_3_ > ⋯, and 0 = *μ*_1_ < *μ*_2_ < *μ*_3_ < ⋯. There is also a set of associated orthogonal left-eigenvectors,

$$\lambda_{k}\;\psi_{k}^{\text{L}}\left(\text{z}\right)=\int d\text{z}\;\psi_{k}^{\text{L}}\left({\text{z}}^{\prime }\right)\;P_{\tau }\left({\text{z}}^{\prime }|\text{z}\right),$$ (12)

with

$$\delta_{kl}=\int d\text{z}\;\psi_{k}^{\text{L}}\left(\text{z}\right)\;\psi_{l}^{\text{R}}\left(\text{z}\right)=\left(\psi_{k}^{\text{L}}\cdot \psi_{l}^{\text{R}}\right)$$ (13)

and $\psi_{k}^{\text{L}}\left(\text{z}\right)=\psi_{k}^{\text{R}}\left(\text{z}\right)\;\rho_{\text{eq}}{\left(\text{z}\right)}^{-1}$. Orthonormalization can be expressed as

$$\delta_{kl}=\int d\text{z}\;\psi_{k}^{\text{L}}\left(\text{z}\right)\;\psi_{l}^{\text{L}}\left(\text{z}\right)\;\rho_{\text{eq}}\left(\text{z}\right).$$ (14)

The first right-eigenvector is actually the equilibrium distribution, $\psi_{1}^{\text{R}}\left(\text{z}\right)=\rho_{\text{eq}}\left(\text{z}\right)$, and the first left-eigenvector is equal to unity. The equilibrium time-correlation function of an arbitrary function *v*(**z**) is

$$\begin{array}{ccc} \left\langle v\left(n\tau \right)\;v\left(0\right)\right\rangle & = & \int d\text{z}\int d{\text{z}}^{\prime }\;v\left({\text{z}}^{\prime }\right)\;P_{n\tau }\left({\text{z}}^{\prime }|\text{z}\right)\;v\left(\text{z}\right)\;\rho_{\text{eq}}\left(\text{z}\right)\\ & = & \underset{k}{\sum }\;{\left(v\cdot \psi_{k}^{\text{R}}\right)}^{2}\;e^{-\mu_{k}n\tau }, \end{array}$$ (15)

where

$$\left(v\cdot \psi_{k}^{\text{R}}\right)=\int d\text{z}\;v\left(\text{z}\right)\;\psi_{k}^{\text{R}}\left(\text{z}\right).$$ (16)

### C. Spectral decomposition of a two state system

Let us return to the normalized population time-correlation function between a “reactant” state *A* and a “product” state *B* of Eq. (2) that is the starting point of the activated-dynamics reactive flux formalism.^1^ The indicator functions, *H*_*A*_ and *H*_*B*_, are defined such that *H*_*A*_ = 1 when the system is in state *A* and zero otherwise and that *H*_*B*_ = 1 when the system is in state *B* and zero otherwise. By construction, *H*_*A*_ + *H*_*B*_ = 1, and the equilibrium probability of the state *A* and *B* is *p*_*A*_ = ⟨*H*_*A*_⟩ and *p*_*B*_ = ⟨*H*_*B*_⟩, respectively.

Relying on a spectral analysis of the Markov dynamics, the long-time relaxation of the system displayed by the equilibrium time-correlation function of the indicator function *H*_*B*_ for the state *B*,

$$\begin{array}{ll} 〈\delta H_{B}\left(0\right)\;\delta H_{B}\left(n\tau \right)〉 & =〈H_{B}\left(0\right)H_{B}\left(n\tau \right)〉-〈H_{B}〉\;〈H_{B}〉\\ & =\int d\text{z}\int d{\text{z}}^{\prime }\;H_{B}\left({\text{z}}^{\prime }\right)\;P_{n\tau }\left({\text{z}}^{\prime }|\text{z}\right)\rho_{\text{eq}}\left(\text{z}\right)\;H_{B}\left(\text{z}\right)\\ & \;-\int d\text{z}\;\rho_{\text{eq}}\left(\text{z}\right)\;H_{B}\left(\text{z}\right)\;\int d{\text{z}}^{\prime }\;\rho_{\text{eq}}\left({\text{z}}^{\prime }\right)\;H_{B}\left({\text{z}}^{\prime }\right)\\ & =\underset{k\geq 1}{\sum }\;{\left(H_{B}\cdot \psi_{k}^{\text{R}}\right)}^{2}\;e^{-\mu_{k}n\tau }-{\left(H_{B}\cdot \psi_{1}^{\text{R}}\right)}^{2}\\ & =\underset{k>1}{\sum }\;{\left(H_{B}\cdot \psi_{k}^{\text{R}}\right)}^{2}\;e^{-\mu_{k}n\tau }, \end{array}$$ (17)

where

$$\left(H_{B}\cdot \psi_{k}^{\text{R}}\right)=\int d\text{z}\;H_{B}\left(\text{z}\right)\;\psi_{k}^{\text{R}}\left(\text{z}\right).$$ (18)

If the indicator function *H*_*B*_ matches the metastable states of the system accurately, the amplitude of the eigenvector $\psi_{2}^{\text{R}}$ should be nearly constant for all the states *i* within the two metastable basins. The integral of the indicator functions with the other eigenvectors should be negligible, i.e., $\left(H_{B}\cdot \psi_{k}^{\text{R}}\right)\approx 0$ for *k* > 2. This means that the second eigenmode $\psi_{2}^{\text{R}}$ essentially represents the global transfer of probability between the metastable basins *A* and *B* and the normalized population time-correlation function from Eq. (2) is

$$\begin{array}{ccc} C\left(n\tau \right) & = & \frac{\underset{k\geq 1}{\sum }\;{\left(H_{B}\cdot \psi_{k}^{\text{R}}\right)}^{2}\;e^{-\mu_{k}n\tau }}{\underset{k>1}{\sum }\;{\left(H_{B}\cdot \psi_{k}^{\text{R}}\right)}^{2}\;}\\ & = & e^{-\mu_{2}n\tau }\;\left(1+\cdots \;\right). \end{array}$$ (19)

Thus, if there is mainly one slow mode corresponding to the transitions between the metastable states *A* and *B*, the long-time relaxation of the correlation function is expected to reflect mainly the second eigenvalue *μ*_2_. In a system with two metastable states, *μ*_2_ can be related to the overall relaxation time corresponding to τ* = 1/*μ*_2_ and the forward and backward transition rates, *k*_*AB*_ = *p*_*B*_ *μ*_2_ and *k*_*BA*_ = *p*_*A*_ *μ*_2_. If the indicator function overlaps with higher order eigenmodes, the dominant relaxation time can be revealed by considering the correlation function over a longer time *τ*_m_ = *nτ*,

$$\begin{array}{ccc} \underset{n\tau \to \tau_{\text{m}}}{\lim}\;\dot{C}\left(n\tau \right) & = & \underset{n\tau \to \tau_{\text{m}}}{\lim}\;\frac{\underset{k\geq 1}{\sum }\;{\left(H_{B}\cdot \psi_{k}^{\text{R}}\right)}^{2}\;\left(-\mu_{k}\right)\;e^{-\mu_{k}n\tau }}{\underset{k>1}{\sum }\;{\left(H_{B}\cdot \psi_{k}^{\text{R}}\right)}^{2}\;}\\ & \approx & -\mu_{2}, \end{array}$$ (20)

which is reasonable as long as $e^{-\mu_{2}\tau_{\text{m}}}\approx 1$ while $e^{-\mu_{3}\tau_{\text{m}}}\ll 1$. This is possible, in practice, only if there is a large gap between the eigenvalues *μ*_2_ and *μ*_3_.

### D. Perspective from transition path theory

#### 1. Steady-state forward flux and committor probability

A spectral analysis may encounter issues when some of the slowest eigenmodes are uncorrelated with the *A* → *B* transition of interest. This issue is circumvented by deliberately selecting the boundary states *A* and *B* to consider the net steady-state reactive flux for the reaction.^7,13–15^ Within the TPT framework,^14^ the net steady-state unidirectional reactive flux from *A* to *B* can be constructed from the probability to make a transition from **z** to **z**′ multiplied by the probabilities that the trajectory arriving at **z** came from *A* and that the trajectory will then go on from **z**′ to reach *B*, minus the probabilities that the trajectory arriving at **z** came from *B* and that the trajectory will then go on from **z**′ to reach *A*. Invoking microscopic detailed balance, this yields

$$J_{AB}=\frac{1}{\tau }\int_{\text{z}\in A^{\prime }}d\text{z}\int_{{\text{z}}^{\prime }\in B^{\prime }}d{\text{z}}^{\prime }\;\left(q\left({\text{z}}^{\prime }\right)-q\left(\text{z}\right)\right)\;P_{\tau }\left({\text{z}}^{\prime }|\text{z}\right)\;\rho_{\text{eq}}\left(\text{z}\right),$$ (21)

where *q*(**z**), called the forward committor, is the probability that a trajectory started at **z** will first reach the state *B*. The bounds on the integral imply that the entire subspace of CVs has been divided in a region *A*′ that includes the state *A* and a region *B*′ that includes the state *B*. For the effective dynamics within the subspace **z**, *q*(**z**) must satisfy the backward propagation condition,

$$q\left(\text{z}\right)=\int d{\text{z}}^{\prime }\;q\left({\text{z}}^{\prime }\right)\;P_{\tau }\left({\text{z}}^{\prime }|\text{z}\right),$$ (22)

with the constraints *q* = 0 when **z** ∈ *A* and *q* = 1 when **z** ∈ *B*. Equation (21) can be derived by counting only the forward flux **z** → **z**′ arising exclusively from reactive *A* → *B* trajectories members of the transition path ensemble^14^ or by considering the net unidirectional reactive flux from *A* to *B* under steady-state conditions.^13^ Because one is free to move the boundary between the regions *A*′ and *B*′, Eq. (21) can be transformed into a convenient unconstrained expression,^12,14,15^

$$\begin{array}{ccc} J_{AB} & = & \frac{1}{2\tau }\int d\text{z}\int d{\text{z}}^{\prime }\;{\left(q\left({\text{z}}^{\prime }\right)-q\left(\text{z}\right)\right)}^{2}\;P_{\tau }\left({\text{z}}^{\prime }|\text{z}\right)\;\rho_{\text{eq}}\left(\text{z}\right)\\ & = & \frac{1}{2\tau }\;\left\langle {\left(q\left(\tau \right)-q\left(0\right)\right)}^{2}\right\rangle \\ & = & \frac{1}{\tau }\;\left(\left\langle q\left(0\right)q\left(0\right)\right\rangle -\left\langle q\left(0\right)q\left(\tau \right)\right\rangle \right)\\ & = & \frac{1}{\tau }\;C_{qq}\left(\tau \right), \end{array}$$ (23)

where we have defined the committor time-correlation function,

$$C_{qq}\left(\tau \right)=〈q\left(0\right)q\left(0\right)〉-〈q\left(0\right)q\left(\tau \right)〉.$$ (24)

Here, *q*(*τ*) is an implicit short-hand notation for $q\left(\tilde{\text{z}}\left[\text{x}\left(\tau \right)\right]\right)$. Equations (23) and (24) can also be used to variationally optimize the committor;^12,14,33–35^ minimizing *J*_*AB*_ with respect to a trial function, i.e., *δJ*_*AB*_[*q*(**z**)]/*δq*(**z**) = 0, recovers the backward propagator condition of Eq. (22). Because the committor to reach state *A* before state *B* is simply equal to (1 − *q*), Eq. (23) makes it clear that the steady-state flux from state *B* to state *A* is equal to that from state *A* to state *B*, *J*_*BA*_ = *J*_*AB*_. The forward transition rate can be determined as *k*_*AB*_ = *J*_*AB*_/*p*_*A*_, and the mean first passage time (MFPT) from *A* to *B* is equal to 1/*k*_*AB*_.

Additional features about reactive paths can be formulated. For example, the probability *p*_*AB*_(**z**) for a *A* → *B* reactive trajectory knowing that the system is at **z** is the probability 1 − *q*(**z**) that the trajectory came from *A* multiplied by the probability *q*(**z**) that the trajectory will lead to *B*. Averaging over all possible **z** weighted by the equilibrium distribution *ρ*_eq_(**z**) yields the total probability of a reactive *A* → *B* trajectory, *p*_*AB*_ = ⟨*q* (1 − *q*)⟩, at equilibrium. The latter may be understood as the fraction of time a very long trajectory of length *t*_tot_ spends on the transition pieces that are purely *A* → *B* reactive, *τ*_r_/*t*_tot_. Since *J*_*AB*_ is the total number of *A* → *B* reactive transitions *n*_r_ during the time *t*_tot_, we can write ⟨*q* (1 − *q*)⟩ = (*τ*_r_/*n*_r_) (*n*_r_/*t*_tot_) = ⟨*τ*_r_⟩ *J*_*AB*_. Thus, the mean transit time during a reactive transition ⟨*τ*_r_⟩ is equal to ⟨*q* (1 − *q*)⟩/*J*_*AB*_.^13,20^ Because *J*_*AB*_ = *J*_*BA*_, then *p*_*AB*_ = *p*_*BA*_ and the mean transit time is independent of the direction.

#### 2. Markovian dynamics in the short time limit

While the present analysis is strictly valid if the effective propagator within the reduced subspace of the CVs is Markovian with the finite lag-time *τ*, one may ask when the reactive flux *J*_*AB*_ can be determined in the limit *τ* → 0,

$$\begin{array}{ccc} J_{AB} & = & \underset{\tau \to 0}{\lim}\;\frac{C_{qq}\left(\tau \right)-C_{qq}\left(0\right)}{\tau }\;\\ & = & {\left.\frac{d}{d\tau }C_{qq}\left(\tau \right)\right|}_{\tau =0} \end{array}$$ (25)

$$\begin{array}{ccc} & = & -\frac{d}{d\tau }{\left.\left\langle q\left(0\right)q\left(t\right)\right\rangle \right|}_{\tau =0}\\ & = & -\int d\text{z}\int d{\text{z}}^{\prime }\;q\left({\text{z}}^{\prime }\right)\;q\left(\text{z}\right)\;\left(\frac{d}{d\tau }{\left.P_{\tau }\left({\text{z}}^{\prime }|\text{z}\right)\right|}_{\tau =0}\right)\;\rho_{\text{eq}}\left(\text{z}\right). \end{array}$$ (26)

The flux is well-defined in the limit of *τ* → 0 if the underlying dynamics arises from a genuine continuous-time Markovian process,

$$\frac{d}{d\tau }{\left.P_{\tau }\left({\text{z}}^{\prime }|\text{z}\right)\right|}_{\tau =0}=K\left({\text{z}}^{\prime }|\text{z}\right),$$ (27)

where K is the transition rate matrix, with $P_{\tau }\equiv e^{K\tau }$ and $P_{0}\left({\text{z}}^{\prime }|\text{z}\right)={\left[e^{0}\right]}_{\left({\text{z}}^{\prime }|\text{z}\right)}=\delta \left(\text{z}-{\text{z}}^{\prime }\right)$, yielding

$$J_{AB}=-\int d\text{z}\int d{\text{z}}^{\prime }\;q\left({\text{z}}^{\prime }\right)\;q\left(\text{z}\right)\;K\left({\text{z}}^{\prime }|\text{z}\right)\;\rho_{\text{eq}}\left(\text{z}\right).$$ (28)

Similarly, the flux is well-defined in the limit of *τ* → 0 if the effective dynamics within the subspace of CVs (**z**) is assumed to be diffusive with diffusion matrix **D**; it can be shown that (Appendix A)

$$J_{AB}=\int d\text{z}\;\nabla q\left(\text{z}\right)\cdot \text{D}\;\rho_{\text{eq}}\left(\text{z}\right)\;\nabla q\left(\text{z}\right).$$ (29)

Under these conditions, the forward reactive flux *J*_*AB*_ is given by the initial slope of the committor correlation function at *τ* = 0, i.e., the time derivative evaluated at *τ* = 0. If should be noted, however, that this expression would be identically equal to zero due to time reversibility if the microscopic dynamics were truly inertial.^36^ Physically, this suggest that the proper limit for *τ* must be larger than 0 to establish the diffusive dynamics, i.e., ${\dot{C}}_{qq}\left(0^{+}\right)\approx \left(C_{qq}\left(\tau \right)-C_{qq}\left(0\right)\right)/\tau$, with a small but finite *τ* > 0.

#### 3. Committor and eigenvectors

It is of interest to relate the unidirectional flux *J*_*AB*_ to the overall relaxation time determined from the spectral analysis. For this, we need to have a model for the committor. While the committor *q*(**z**) is not quite a left-eigenvector of the effective propagator, a useful approximate construction can be written as^37^

$$q\left(\text{z}\right)\approx -\left(\frac{a}{b-a}\right)\;\psi_{1}^{\text{L}}\left(\text{z}\right)+\left(\frac{1}{b-a}\right)\;\psi_{2}^{\text{L}}\left(\text{z}\right),$$ (30)

where $a=\psi_{2}^{\text{L}}\left(\text{z}\right)$ with **z** ∈ *A* and $b=\psi_{2}^{\text{L}}\left(\text{z}\right)$ with **z** ∈ *B*. The constants *a* and *b* must have opposite sign to guarantee that the vector is orthogonal to the equilibrium vector. It can be shown (Appendix B) that *a* = −(*b* − *a*) *p*_*B*_, and $1={\left(b-a\right)}^{2}\;\left(p_{A}\;p_{B}-\left\langle q\left(1-q\right)\right\rangle \right)$.

This construction makes a function *q*(**z**) that is equal to 0 for **z** ∈ *A* and equal to 1 for **z** ∈ *B*, which approximately satisfies the backward propagation condition of Eq. (22). Using the model committor, it can be shown (Appendix C) that the net unidirectional reactive flux from *A* to *B* is

$$J_{AB}=p_{A}\;p_{B}\;\frac{1}{\tau }\;\left(1-e^{-\mu_{2}\tau }\right)\;{\left(1+\frac{\left\langle \tau_{\text{r}}\right\rangle }{\tau }\left(1-e^{-\mu_{2}\tau }\right)\right)}^{-1}.$$ (31)

This expression is the steady-state flux from *A* to *B* for the simple two-state model. For this result to be valid, the lag-time *τ* must be sufficiently long to yield an effective propagation within the subspace **z** that is Markovian. If this condition is met, then the net forward flux *J*_*AB*_ is independent of the value of the lag-time. If we assume that *μ*_2_*τ* ≪ 1, we have $\left(e^{-\mu_{2}\tau }-1\right)\approx \left(1-\mu_{2}\tau +\cdots -1\right)$ and that ⟨*τ*_r_⟩ *μ*_2_ ≪ 1, then we have *J*_*AB*_ ≈ *p*_*A*_ *p*_*B*_*μ*_2_ = *p*_*A*_ *k*_*AB*_. The expression shows that the steady-state flux from *A* to *B* remains the same if the lag-time is *nτ*, as long as *nμ*_2_*τ* ≪ 1.

#### 4. Approximate committors

The formal analysis relies on the existence of the effective propagator $P_{\tau }\left({\text{z}}^{\prime }|\text{z}\right)$ representing the Markovian dynamics of the system within the subspace of the CVs, **z**. Nonetheless, it is understood that the microscopic trajectory **x**(*t*) is generated from the elementary propagator *P*_Δ*t*_(**u**′|**u**) with a time step Δ*t* that may be much shorter than the lag-time *τ*. Formally, one can always fall back on the elementary propagator to represent the time-evolution of the system. In this context, the broadest perspective is offered by treating the function *q*(**z**) as a function of the microscopic configuration **x**(*t*) that can be evaluated at any arbitrary time *t*, i.e., $q\left(\tilde{\text{z}}\left[\text{x}\left(t\right)\right]\right)$. The time-correlation function *C*_*qq*_(*t*) may be considered, even if the underlying dynamics is non-Markovian and comprises inertial aspects at times shorter than *τ* (this will be the case in Fig. 3). Obviously, the function *q*(**z**) associated with a Markovian dynamics within the subspace of the CVs is only an approximation to the committor *q*(**x**, **v**) in the full phase space. However, this construct does nonetheless provide a useful framework to analyze the true microscopic dynamics of the system. Importantly, the validity of the final rate expression does not require our representation of the effective dynamics within the subspace of the CVs to be exact at all times. A similar strategy was used by Ruiz-Montero *et al.*^32^ to formulate an efficiency activated-dynamics reactive flux simulations for systems experiencing large dissipative forces (we return to this in Sec. IV).

If the dynamics of the CVs genuinely corresponds to a diffusion process or is governed by a continuous-time Markov process, then the committor time-correlation function *C*_*qq*_(*t*) is rigorously linear for *t* > 0^+^. In this case, there is no short transient at small times and the committor time-correlation function is immediately linear. The forward transition rate follows directly from the forward unidirectional flux *J*_*AB*_ in Eq. (31). Whenever any of those conditions are not satisfied, there will be some transient behavior at short time and the variational forward flux relation will be valid only for a lag-time that is sufficient long to establish the linear behavior of *C*_*qq*_(*t*). For example, the forward flux relation is not valid if the dynamical propagation within the subspace **z** is non-Markovian because the lag-time *τ* is too short or if an approximate committor function *q*_⋆_(**z**) is used. In this case, the equilibrium time-correlation function from Eq. (15) is

$$\begin{array}{ccc} \left\langle q_{⋆}\left(0\right)\;q_{⋆}\left(\tau \right)\right\rangle & = & \int d\text{z}\int d{\text{z}}^{\prime }\;q_{⋆}\left(\text{z}\right)\;q_{⋆}\left({\text{z}}^{\prime }\right)\;P_{\tau }\left({\text{z}}^{\prime }|\text{z}\right)\;\rho_{\text{eq}}\left(\text{z}\right)\\ & = & \underset{k}{\sum }\;{\left(q_{⋆}\cdot \psi_{k}^{\text{R}}\right)}^{2}\;e^{-\mu_{k}\tau }, \end{array}$$ (32)

where

$$\left(q_{⋆}\cdot \psi_{k}^{\text{R}}\right)=\int d\text{z}\;q_{⋆}\left(\text{z}\right)\;\psi_{k}^{\text{R}}\left(\text{z}\right).$$ (33)

Such an approximate committor function may overlap with higher order eigenvectors, $\psi_{k}^{\text{L}}$ with *k* > 2. The correlation function will relax to the dominant rate if some lag-time *τ*_m_ exists such that $e^{-\mu_{3}\tau_{\text{m}}}\ll 1$ while $e^{-\mu_{2}\tau_{\text{m}}}\approx 1$. In this case, the committor time-correlation function is not immediately linear, even if the dynamics within the subspace **z** is Markovian. As *t* increases, *C*_*qq*_(*t*) should begin to vary linearly with time, a behavior that is valid only up to a certain time scale because $C_{qq}\left(t\right)\to \left\langle {\left(q_{⋆}-\left\langle q_{⋆}\right\rangle \right)}^{2}\right\rangle$ in the limit *t* → *∞*. There is an intermediate finite time scale *τ*_m_ larger than the Markovian lag-time *τ* where the correlation function may be expected to vary linearly with time. Because of the expected linear dependence, the net forward unidirectional flux *J*_*AB*_ is essentially given by the slope “s” of the correlation function *C*_*qq*_(*t*). This argument is valid if the committor time-correlation function has the form *C*_*qq*_(*t*) = *c*(*t*) + *st*, where *c*(*t*) is a small component that rapidly decays to some finite plateau value with $\underset{t\to \tau_{\text{m}}}{\lim}\;\dot{c}\left(t\right)=0$. Accordingly, we can write that the limiting slope of the committor time-correlation function is given by

$$J_{AB}=\underset{t\to \tau_{\text{m}}}{\lim}\;{\dot{C}}_{qq}\left(t\right).$$ (34)

The microscopic time *τ*_m_ required for this relation to be valid may need to be longer than the lag-time *τ* that is needed to yield a Markovian dynamics within the subspace **z**.

## III. ILLUSTRATIVE SIMULATIONS

**FIG. 1.**
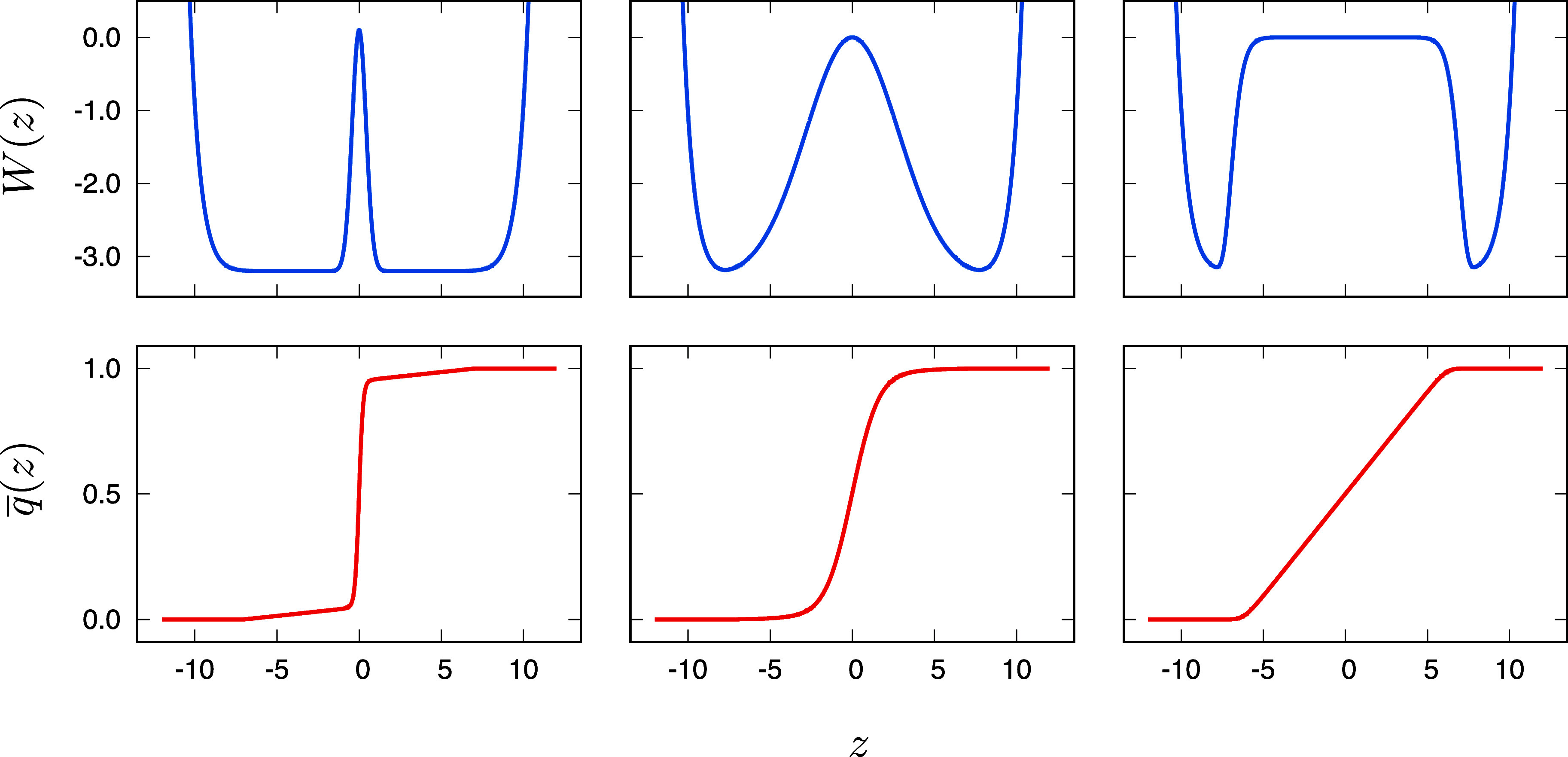
Schematic illustration of a two-state system comprising a single narrow, medium, and broad free energy barrier. At the top (in blue), the potentials *W*(*z*) are defined via Eq. (35) in kcal/mol as a function of *z* in Å. In the bottom (in red), the committors *q*(*z*) for the different potentials are calculated via Eq. (37) with *z*_1_ and *z*_2_ set to −7 and +7, respectively.

For illustrative purposes, we consider three simple double-well one-dimensional systems with the potential,

$$W\left(z\right)=\left\{\begin{array}{cc} {\left(\frac{z}{9.5}\right)}^{12}+3.3\;e^{\left(-{\left(z/0.6\right)}^{2}\right)}-3.2\; & \text{“narrow,”}\\ {\left(\frac{z}{9.5}\right)}^{12}+3.3\;e^{\left(-{\left(z/4.0\right)}^{2}\right)}-3.3\; & \text{“medium,”}\;\;\;\;\;\\ {\left(\frac{z}{9.5}\right)}^{12}+3.2\;e^{\left(-{\left(z/7.0\right)}^{16}\right)}-3.2\; & \text{“broad”}, \end{array}\right.\;$$ (35)

where *z* is in Å and *W*(*z*) is in kcal/mol. The potentials are shown in Fig. 1 (top). The symmetric wells yield equilibrium probabilities *p*_*A*_ = *p*_*B*_ = 0.5. In all cases, the double-well system is symmetric with a single free energy barrier, which, in turn, can be characterized as “narrow,” “medium,” and “broad.” The Euler–Lagrange equation determining the committor probability *q*(*z*) is

$$\frac{\partial }{\partial z}\;\left(e^{-W\left(z\right)/k_{\text{B}}T}\;\frac{\partial q\left(z\right)}{\partial z}\right)=0,$$ (36)

subject to the constraint ${\left.q\left(z\right)\right|}_{z\leq z_{1}}=0$ and ${\left.q\left(z\right)\right|}_{z\geq z_{2}}=1$. The solution is

$$q\left(z\right)=\frac{\int_{z_{1}}^{z}\;e^{W\left(z^{\prime }\right)/k_{\text{B}}T}dz^{\prime }}{\int_{z_{1}}^{z_{2}}\;e^{W\left(z^{\prime }\right)/k_{\text{B}}T}dz^{\prime }},$$ (37)

where *k*_B_*T* = 0.5915 kcal/mol. For the sake of simplicity, the boundaries *z*_1_ and *z*_2_ were set to −7 and +7, respectively, for the three cases. The calculated committors are shown in Fig. 1 (bottom). Each system was simulated with Brownian dynamics (BD),^30^ assuming a diffusion coefficient *D* = 1 Å^2^ ps^−1^. A time step of 0.005 ps was used to generate a 1 *µ*s trajectory (200 × 10^6^ steps). Because the BD trajectory is Markovian along the *z* axis, the time step of 0.005 ps is the same as the lag-time *τ*.

The system with a broad barrier was also simulated with Langevin dynamics,^30^ assuming a diffusion coefficient *D* = 1 Å^2^/ps and a mass m of 20 atomic-mass-unit. For these parameters, the relaxation time of the velocity–velocity correlation function *k*_B_*T*/*Dm* is 0.08 ps. At this time scale, the Langevin dynamics becomes essentially equivalent to a Brownian dynamics within a short time *τ*. A time step of 0.001 ps was used to generate a 1 *µ*s trajectory (10^9^ steps).

Three types of time-correlation functions were calculated from the BD trajectories. First, we consider the committor time-correlation function *C*_*qq*_(*t*) = ⟨*q*(0) *q*(0)⟩ − ⟨*q*(0) *q*(*t*)⟩, which was defined in Eq. (24). We also consider the indicator time-correlation function *C*_*BB*_(*t*) defined as

$$\begin{array}{ccc} C_{BB}\left(t\right) & = & p_{A}\;p_{B}\;\left(1-\frac{〈\delta H_{B}\left(0\right)\;\delta H_{B}\left(t\right)〉}{〈\delta H_{B}\left(0\right)\;\delta H_{B}\left(0\right)〉}\right)\\ & = & 〈H_{B}\left(0\right)\;H_{B}\left(0\right)〉-〈H_{B}\left(0\right)\;H_{B}\left(t\right)〉. \end{array}$$ (38)

The ratio in the parentheses is the normalized population time-correlation function introduced in Eq. (2) to develop the reactive flux formulation. It is easy to show that the forward flux $J_{AB}=p_{A}\;k_{AB}=\underset{t\to \tau_{\text{m}}}{\lim}{\dot{C}}_{BB}\left(t\right)$. Because *C*_*BB*_(*t*) is very similar in appearance to the committor-based time-correlation function *C*_*qq*_(*t*), this form is more convenient to compare the two different formulations. Finally, we define the position time-correlation function as

$$C_{zz}\left(t\right)=p_{A}\;p_{B}\;\left(1-\frac{〈\delta z\left(0\right)\;\delta z\left(t\right)〉}{〈\delta z\left(0\right)\;\delta z\left(0\right)〉}\right),$$ (39)

with *δz*(*t*) = *z*(*t*) − ⟨*z*⟩. By construction, we have *C*_*BB*_(*t*) = *C*_*zz*_(*t*) = *C*_*qq*_(*t*) = 0 at *t* = 0, and all three time-correlation functions are expected to converge to a straight line with the same limiting slope as *t* increases (but remains much smaller than τ*). The time derivative of *C*_*qq*_(*t*) evaluated at the lag-time *τ* is related to the steady-state reactive flux, *J*_*AB*_ according to Eq. (23). Following Eq. (4), the time derivative ${\dot{C}}_{BB}\left(t\right)$ in the limit of *t* → *τ*_m_ also yields the steady-state reactive flux, although the molecular time scale *τ*_m_ may be longer than the lag-time *τ*. Finally, according to Eq. (15), the time derivative of ${\dot{C}}_{zz}\left(t\right)$ is expected to behave similarly in the long time limit.

## IV. DISCUSSION

### A. Reactive flux and spectral analysis

The activated-dynamics reactive flux time-correlation function of Eq. (4) must decay to a plateau value within a molecular time scale *τ*_m_ for the transition rate of the system to be well defined.^1,5,6^ Central to this argument is the assumption that the normalized population time-correlation function of Eq. (2) follows a simple exponential decay with the overall relaxation time τ* for times *t* ≫ *τ*_m_. The spectral analysis of the effective propagator $P_{\tau }\left({\text{z}}^{\prime }|\text{z}\right)$ in terms of its eigenvalues *μ*_*k*_ leading to Eq. (20) clarifies the context supporting this assumption. Relating the activated-dynamics to the spectral analysis by comparing Eqs. (2) and (20), we can see that to properly extract the overall relaxation time of the two-state system, *τ*_m_ must be chosen such that the conditions, *μ*_2_*τ*_m_ ≪ 1 and *μ*_3_*τ*_m_ ≫ 1, are satisfied. This is possible only if there exists a large gap in the eigenvalue spectrum, *μ*_2_ ≪ *μ*_3_. This is a requirement that the two-state system must satisfy; otherwise, the theory based on the fluctuations expressed in Eq. (2) is inappropriate.

The spectral analysis also shed some light on important aspects of the activated-dynamics reactive flux algorithm to efficiently determine a transition rate from computer simulations. In practice, it is well understood that the algorithm is very efficient when the two states are separated by a sharp free energy barrier, the reactive flux time-correlation function in Eq. (4) rapidly within a relatively short time *τ*_m_. However, it is also recognized that the algorithm may not be as effective in the case of slow diffusive motion over a broad flat free energy barrier. In this case, a much longer time *τ*_m_ may be needed to reach the plateau of the reactive flux time-correlation function, requiring the simulation of long activated trajectories.

### B. Illustrative simulations

**FIG. 2.**
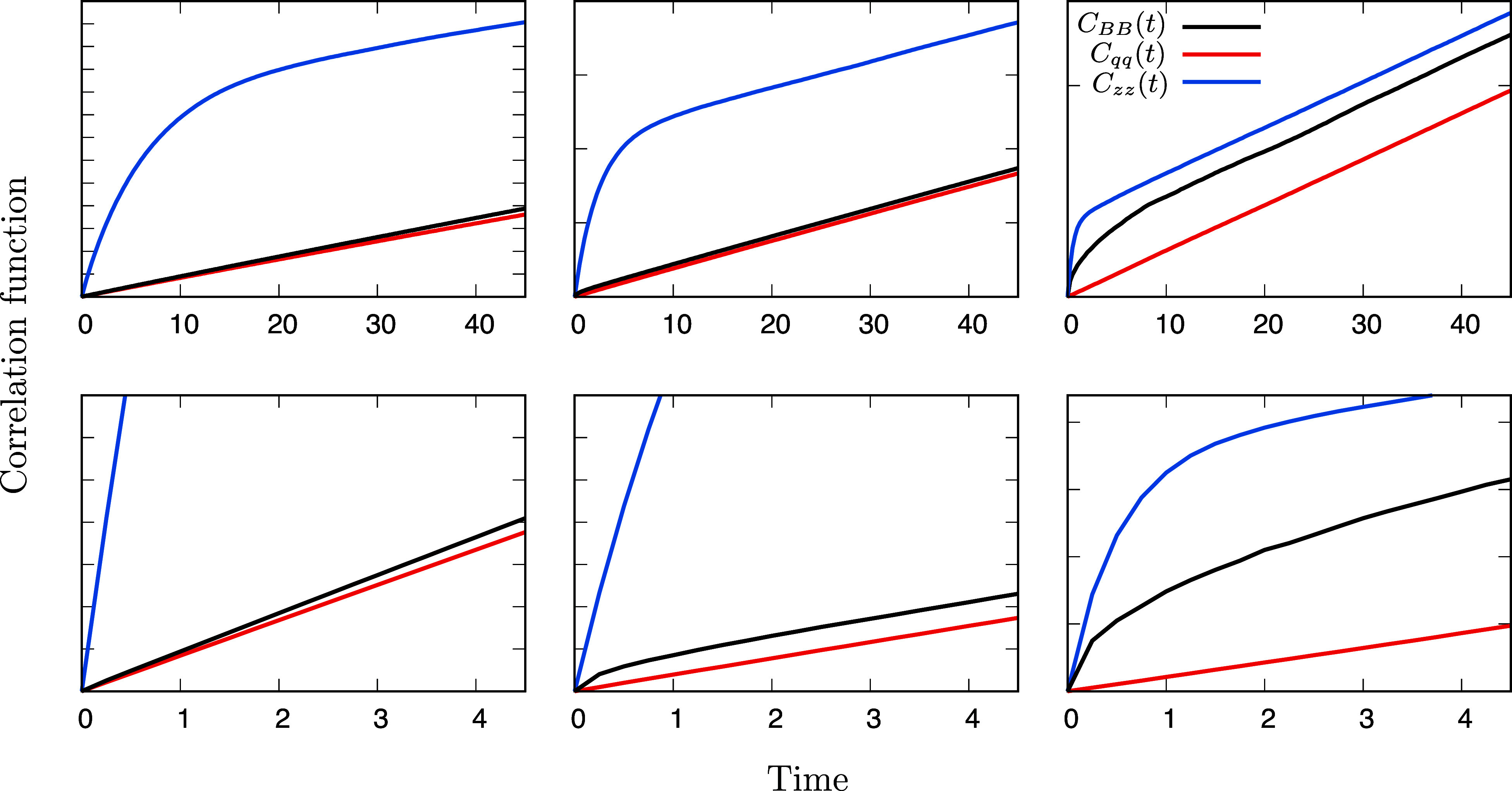
Time-correlation functions calculated from Brownian dynamics simulations for the simple two-state system with a narrow, medium, and broad free energy barrier (shown in Fig. 1). Shown is the indicator autocorrelation function *C*_*BB*_(*t*) (in black), the committor correlation function *C*_*qq*_(*t*) (in red), and the position autocorrelation function *C*_*zz*_(*t*) (in blue). The bottom plots show a zoom of the plots at the top. For a given potential, all the correlation functions converges to the same slope, which is related to the net steady-state unidirectional reactive flux *J*_*AB*_. For a narrow, medium, and broad free energy barrier, *J*_*AB*_ is estimated to be 4.4 × 10^−4^, 1.9 × 10^−4^, and 1.1 × 10^−4^ ps^−1^, respectively. The tick marks along the *y*-axis are evenly separated by 0.005 in the top plot and by 0.0005 in the bottom plot (the *y* labels are not displayed for the sake of clarity). The time axis is in ps.

To illustrate these points, Brownian dynamics simulations were carried out for simple one-dimensional systems (Fig. 1). The main results are shown in Fig. 2. The indicator time-correlation function *C*_*BB*_(*t*) is related to the activated-dynamics formalism to calculate the transition rate.^1^ Position time-correlation functions, such as *C*_*zz*_(*t*), appear in the time-lagged independent component analysis (TICA).^38,39^ The committor time-correlation function *C*_*qq*_(*t*) originate from the TPT framework.^7,12,14,15,33,40^ From Fig. 2, it is clear that all time-correlation functions ultimately converge to the same slope, reflecting the global relaxation time within the system. However, the different time-correlation functions reach the same slope differently. When the barrier is extremely narrow, the indicator time-correlation function *C*_*BB*_(*t*) converges extremely rapidly to a straight line with constant slope, whereas the position autocorrelation function *C*_*zz*_(*t*) takes a longer time. There are large fluctuations along *z* in the two broad wells, but those fluctuations are non-reactive—they are not associated with a *A* → *B* transition. In contrast, the fluctuations in *C*_*BB*_ are faithfully reporting on such transition. In contrast, when the barrier is broad, the position autocorrelation function *C*_*zz*_(*t*) converges to a straight line more rapidly, whereas the indicator time-correlation function *C*_*BB*_(*t*) takes a much a longer time. Nonetheless, even in the case of the broad barrier, the time-correlation functions relaxes to its constant slope within less than 1–2 ps. This is shorter than the time needed to diffuse from the center of the barrier to its edge, estimated as 2*Dt* = *L*^2^, with *D* = 1 Å^2^ ps^−1^ and *L* = 7 Å, to be on the order of 25 ps. The latter time scale is comparable to the mean transit time during a reactive transition, ⟨*τ*_r_⟩ = ⟨*q* (1 − *q*)⟩/*J*_*AB*_,^13,20^ which is estimated to be on the order of 25 ps in this case. In the present simulations, *J*_*AB*_ is estimated to be 4.4 × 10^−4^, 1.9 × 10^−4^, and 1.1 × 10^−4^ ps^−1^ for the narrow, medium, and broad barrier, respectively. The corresponding global relaxation times $\tau^{*}={\left(\mu_{2}\right)}^{-1}$ are estimated to be 568, 1315, and 2272 ps, which is much longer than the Markovian lag-time, *τ* (0.005 ps), and the mean transit time of reactive transitions, ⟨*τ*_r_⟩.

### C. Reactive flux and approximate committor

The activated-dynamics reactive flux formalism requires the definition of a dividing surface between the states *A* and *B*, whereas the TPT framework requires the definition of two boundary regions associated with the states *A* and *B*. Yet, it is interesting to note that the expressions derived above for the forward transition rate *k*_*AB*_ from the activated-dynamics reactive flux formalism bears a certain similarity to that from the committor time-correlation function. Equations (4) and (34) are equivalent if one imagines that the indicator function *H*_*B*_ is substituted for the committor *q*. Specifically, we have that ${\dot{C}}_{qq}\left(\tau \right)={\dot{C}}_{BB}\left(\tau_{\text{m}}\right)=J_{AB}$, with the substitution $\left\langle q\left(0\right)\dot{q}\left(t\right)\right\rangle \to \left\langle H_{B}\left(0\right){\dot{H}}_{B}\left(t\right)\right\rangle$. Like the committor *q*, *H*_*B*_ is equal to zero in state *A* and one in state *B*, and in that sense, the Heaviside step-function *H*_*B*_ stands as some kind of crude approximation to the exact committor. By extension, when there are multiple collective variables **z**, one could imagine defining the reaction coordinate as *x* = (**z** − **z**^†^) · ***n*** and the indicator function as

$$H_{B}\left(x\right)=\theta \left(\left(\text{z}-{\text{z}}^{†}\right)\cdot n\right).$$ (40)

For *H*_*B*_(*x*) to serve as an effective (albeit crude) approximation to the committor *q*_⋆_(**z**), the vector ***n*** should be parallel to the gradient of the committor, ***∇****q*(**z**^†^), with **z**^†^ in the vicinity of the transition state region. For example, **z**^†^ can be defined as

$${\text{z}}^{†}=\frac{\int d\text{z}\;\text{z}\;\delta \left(q\left(\text{z}\right)-0.5\right)\rho_{\text{eq}}\left(\text{z}\right)}{\int d\text{z}\;\delta \left(q\left(\text{z}\right)-0.5\right)\rho_{\text{eq}}\left(\text{z}\right)},$$ (41)

where *q*(**z**) = 0.5 is the separatrix. Because the indicator function in Eq. (40) serves as an approximate committor *q*_⋆_(**z**), a time *τ*_m_ longer than *τ* may be needed in this case to reach the linear regime of the time-correlation function. It is also interesting to consider Eq. (40) as a functional form depending parametrically on ***n*** and **z**^†^. Recalling that Eqs. (23) and (24) for the reactive flux *J*_*AB*_ and time-correlation function *C*_*qq*_(*τ*) provide a variational principle to determine the committor from a trial function,^12,14,33–35^ one would minimize the reactive flux *J*_*AB*_ as a function of ***n*** and **z**^†^ to obtain variationally optimized parameters. This sheds new light on the significance of variational TST, which seeks to optimize the reaction coordinate by minimizing the transition rate.^41^ It also suggests that the most meaningful reactive direction at the transition state in the multidimensional space of the CVs is parallel to ***∇****q*(**z**^†^), consistent with the multidimensional Kramers–Langer theory.^42^ Indeed, as noted in TPS studies,^17^ configurations sampled according to $\delta \left.\left(\text{z}-{\text{z}}^{†}\right)\cdot n\right)\;\rho_{\text{eq}}\left(\text{z}\right)$ lead to a broad a distribution of committor probabilities when using a suboptimal reaction coordinate with a vector ***n*** that is not parallel to ***∇****q*(**z**^†^).

Approximating the committor as a Heaviside step-function is likely to be more accurate when a sharp free energy barrier separates the states *A* and *B*. However, in the case of diffusive dynamics occurring on the top of a wide flat barrier, such a Heaviside step-function is not a very good representation of the correct committor. This is precisely the situation where the activated-dynamics reactive flux algorithm encounters difficulties to converge rapidly (Fig. 2). This suggest that designing improved model committors may help formulate more efficient computational algorithms to determine the transition rate.

The idea of exploiting an approximate committor as a framework to improve the convergence of a transition rate calculation bares a certain similarity with a very interesting strategy previously designed by Ruiz-Montero, Frenkel, and Brey.^32^ Their method, which also starts from the idea that the Heaviside step functions in the high friction limit should be replaced with the smooth function akin to the committor, was shown to dramatically improve the statistical convergence of the reactive flux algorithm for diffusive barriers. While they used an initial perturbation and characteristic functions that resemble the steady state concentration profile, it is well known in TPT that the latter equal to $\rho_{\text{eq}}\left(\text{z}\right)q\left(\text{z}\right)\left(1-q\left(\text{z}\right)\right)$.^13^ As with the idea of using an approximate committor, the high friction steady-state picture served only to better formulate the problem in their method, and the validity of the final rate expression did not require this picture to be exact.^32^

### D. Committor as reaction coordinate

A striking observation from Fig. 2 is that the committor time-correlation function *C*_*qq*_(*t*) yields the correct slope at very short time in all cases, independent of the shape of the free energy barrier. This is in strong contrast with the time-correlation functions *C*_*BB*_(*t*) and *C*_*zz*_(*t*). The convergence of the committor time-correlation function to the correct slope within the lag-time *τ* is expected. If the underlying dynamics in the subspace **z** is governed by a continuous-time Markov process as in Eq. (28) or by a diffusion process as in Eq. (A1), then the committor time-correlation function is immediately linear and the transition rate can be determined in the limit of *t* → 0^+^ from Eq. (28). When those conditions are not quite satisfied, there will be some transient behavior over the time-lag *τ* needed to achieve Markovity for the effective propagator $P_{\tau }$ within the subspace **z**.^12^ Establishing the Markovity of $P_{\tau }$ is generally non-trivial, although the present observations suggest that a simple criteria might be to verify whether the time derivative ${\dot{C}}_{qq}\left(t\right)$ reaches a plateau as *t* → *τ*. Whether this conjecture can be demonstrated rigorously is unclear. Once this is done, the net steady-state reactive flux *J*_*AB*_ and the transition rate *k*_*AB*_ evaluated at the lag-time *τ* can be obtained on the basis of Eq. (23).

It is sometimes tempting to imagine that the dynamics along the committor is Markovian with the lag-time *τ*. To elaborate on this point, *C*_*qq*_ could be formally expressed as

$$C_{qq}\left(\tau \right)=\frac{1}{2}\;\int dq\int dq^{\prime }\;{\left(q^{\prime }-q\right)}^{2}\;P_{\tau }\left(q^{\prime }|q\right)\;\rho_{\text{eq}}\left(q\right),$$ (42)

where $P_{\tau }\left(q^{\prime }|q\right)$ represents an effective reduced propagator for the forward step (*q* → *q*′) for the time step *τ*, which can be defined via Eqs. (9) and (10). Let us recall that *τ* is the lag-time that achieves Markovity for the effective propagator $P_{\tau }$ within the subspace **z**.^12^ However, there is no guaranty that the dynamics of the system projected onto the one-dimensional coordinate *q* ought to be Markovian with the same lag-time *τ*. Therefore, while Eq. (42) is rigorously correct, it does not imply that the effective propagator $P_{\tau }\left(q^{\prime }|q\right)$ obeys the Chapman–Kolmogorov equation. In fact, achieving a self-consistent representation of the dynamics of the system projected onto the committor with the lag-time *τ* (closure of the dynamical propagation) is likely to require non-Markovian memory effects.^43–47^

Nonetheless, there are reasons to believe that the committor might serve as a useful guide to define a reaction coordinate. Indeed, it has been shown that in the case of a multidimensional activated process controlled by diffusion, using the vector normal to the isocommittor plane separatrix (*q*^†^ = 0.5) to construct a one-dimension reaction coordinate yields a rate constant that is identical to that predicted by the multidimensional Kramers–Langer theory.^42^ Similarly, as made clear by Eq. (40), the most effective 1D reaction coordinate in a multidimensional space **z** is parallel to the gradient of the committor in the region of the transition state, ***∇****q*(**z**^†^). However, simply treating the committor as a one-dimensional reaction coordinate in the activated-dynamics formalism does not necessarily alleviate the issues caused by slow relaxation and intermediate time scales. In practice, redefining the population operator *H*_*B*_ on the basis of the separatrix *q*^†^ would return the same normalized population time-correlation function from Eq. (2) and the same reactive flux time-correlation function. In this case, the committor-based reactive flux of Eq. (45) will converge more rapidly than the activated-dynamics reactive flux time-correlation function Eq. (4).

### E. Indicator-restricted committor time-correlation function

One of the most attractive features of the activated-dynamics reactive flux formalism as expressed by Eq. (4) is that it is based on a local sampling with trajectories initiated at the transition state, *x*^†^, along some one-dimensional reaction coordinate.^1,5,6^ This is in contrast with the reactive flux expression based on the committor time-correlation function Eq. (23), which is based on an unconstrained equilibrium average over all possible initial positions within the subspace **z**. Seeking to recover such a local sampling, we consider the steady-state reactive flux from *A* to *B* based on Eq. (21) expressed in terms of transitions from the point **z** with committor *q*(**z**) < *q*^†^ to the point **z**′ with committor *q*(**z**′) > *q*^†^,

$$\begin{array}{ccc} J_{AB}^{†} & = & \frac{1}{\tau }\;\int d\text{z}\int d{\text{z}}^{\prime }\;\theta \left(q^{†}-q\left(\text{z}\right)\right)\;\theta \left(q\left({\text{z}}^{\prime }\right)-q^{†}\right)\\ & & \times \left(q\left({\text{z}}^{\prime }\right)-q\left(\text{z}\right)\right)\;P_{\tau }\left({\text{z}}^{\prime }|\text{z}\right)\;\rho_{\text{eq}}\left(\text{z}\right)\\ & = & \frac{1}{\tau }\;\left\langle \theta \left(q\left(\tau \right)-q^{†}\right)\;\theta \left.\left(q^{†}-q\left(0\right)\right)\right)\left(q\left(\tau \right)-q\left(0\right)\right)\right\rangle . \end{array}$$ (43)

This expression is formally correct for any value of 0 < *q*^†^ < 1. For the sake of convenience, we choose the separatrix where *q*^†^ = 0.5. By identification, we can then write the state indicator functions as $\theta \left(q-q^{†}\right)=H_{A}$ and $\theta \left(q^{†}-q\right)=H_{B}$. We follow similar steps from Eq. (4), leading to the development of the activated-dynamics reactive flux formalism. Defining the indicator-restricted committor time-correlation function,

$$C_{AB‐qq}\left(t\right)=\left\langle H_{A}\left(0\right)\;H_{B}\left(t\right)\;\left(q\left(t\right)-q\left(0\right)\right)\right\rangle ,$$ (44)

the reactive flux at the boundary between the *A* and *B* regions can be written as

$$\begin{array}{ccc} J_{AB}^{†} & = & \underset{t\to \tau }{\lim}\;{\dot{C}}_{AB‐qq}\left(t\right)\\ & = & \underset{t\to \tau }{\lim}\;\left\langle H_{A}\left(0\right)\;\frac{d}{dt}\left[H_{B}\left(t\right)\;\left(q\left(t\right)-q\left(0\right)\right)\right]\right\rangle \\ & = & -\underset{t\to \tau }{\lim}\;\left\langle {\dot{H}}_{A}\left(0\right)\;H_{B}\left(t\right)\;\;\left(q\left(t\right)-q\left(0\right)\right)\right\rangle \\ & = & \left\langle \left.\delta \left(q\left(0\right)-q^{†}\right)\right)\;\dot{q}\left(0\right)\;H_{B}\left(\tau \right)\;\left(q\left(\tau \right)-q\left(0\right)\right)\right\rangle . \end{array}$$ (45)

Like the activated-dynamics reactive flux formalism of Eq. (4), this expressions is based on a local sampling of trajectories initiated at a given interface, with $q\left(\tilde{\text{z}}\left[\text{x}\right]\right)=q^{†}$. From this point of view, the expression focuses the calculation of the forward transition rate constant at a transition state. By analogy with activated-dynamics, one may note that the forward rate in Eq. (4) could also be expressed from the flux at the interface between the state *A* and *B* as $k_{AB}=\left\langle H_{A}\left(0\right){\dot{H}}_{B}\left(\tau_{\text{m}}\right)\right\rangle /p_{A}$. However, it is noteworthy that this form is not equivalent to simply substituting *x* → *q* in Eq. (4). Equation (45) is not the same as simply using the committor as a “reaction coordinate” in the reactive flux formalism. What is different is the term [q(τ)−q(0)], weighting differently the dynamical excursion away from the dividing surface between the states *A* and *B*, which greatly affects the convergence of the reactive flux. This committor-based term is present here because the fundamental expression for the reactive flux at the boundary between the *A* and *B* regions Eq. (21) is derived by including only local transitions **z** → **z**′ that belong to reactive paths with genuine *A* → *B* transitions.^12^

**FIG. 3.**
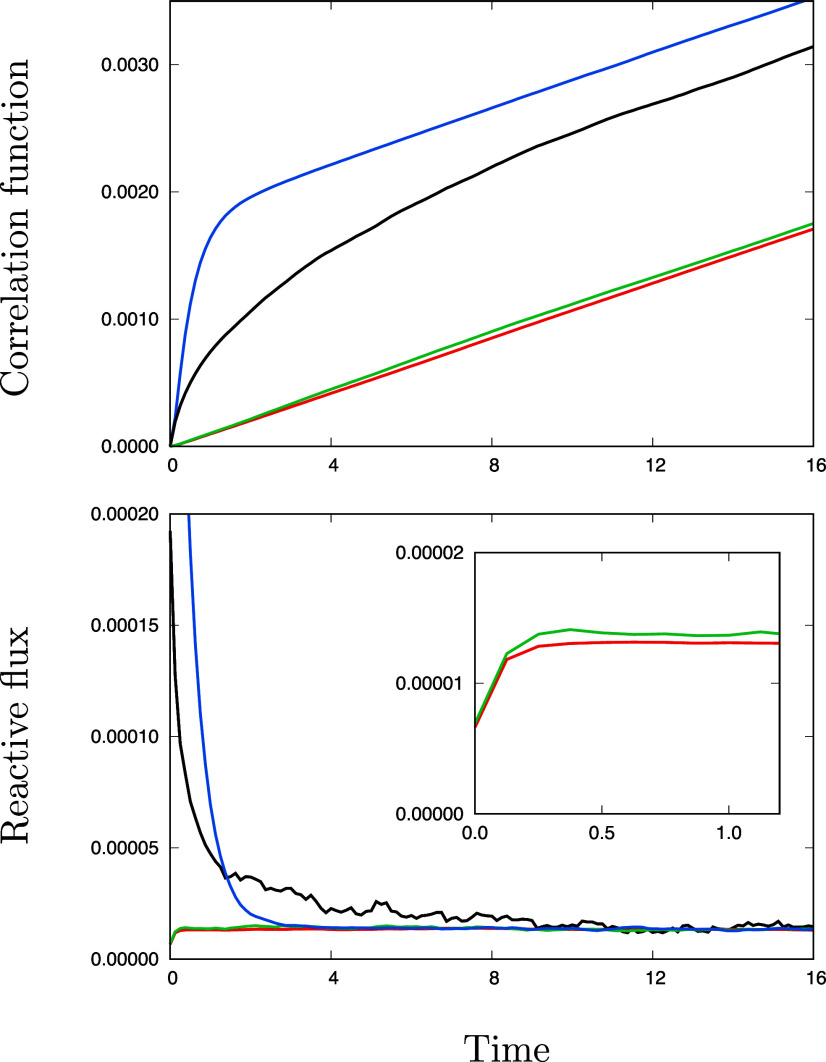
Time-correlation functions for the simple two-state system with a broad free energy barrier (shown in Fig. 1). In the top, the indicator autocorrelation function *C*_*BB*_(*t*) (in black), the position autocorrelation function *C*_*zz*_(*t*) (in blue), the committor time-correlation function *C*_*qq*_(*t*) (in red), and the indicator-restricted committor time-correlation function *C*_*AB*-*qq*_(*t*) (in green) is shown. The time axis is in ps. All the time-correlation functions converge to the same slope, which is related to the steady-state forward reactive flux *J*_*AB*_ (top). The time-derivative of the time-correlation functions yielding the reactive flux are plotted (bottom) to illustrate how the slope relaxes to a constant plateau value corresponding to *J*_*AB*_ = *p*_*A*_ *k*_*AB*_ = 1.05 × 10^−4^ ps^−1^, close to the value for the broad barrier obtained by Brownian dynamics reported in Fig. 2.

To illustrate these ideas and further examine the convergence of the activated-dynamics reactive flux formalism and transition state theory,^1,5,6^ we consider again the time-correlation functions calculated from Langevin simulations in the case of the broad free energy barrier. The parameters were chosen such that the Langevin dynamics is in a high friction limit and becomes equivalent to Brownian dynamics within the short time-lag *τ* = 0.08 ps. The time-correlation functions are shown in Fig. 3. The indicator function time-correlation function *C*_*BB*_(*t*) and its time-derivative ${\dot{C}}_{BB}\left(t\right)$ corresponding to the reactive flux are shown at the top and bottom, respectively. The initial value of ${\dot{C}}_{BB}\left(t\right)$ evaluated at *t* = 0^+^ corresponds to *p*_*A*_ times the transition state rate $k_{AB}^{\text{TST}}$. The plateau value of ${\dot{C}}_{BB}\left(\tau_{\text{m}}\right)$ is equal to *p*_*A*_ times the forward transition rate *k*_*AB*_, and the transmission coefficient *κ*(*τ*_m_) is equal to ${\dot{C}}_{BB}\left(\tau_{\text{m}}\right)/{\dot{C}}_{BB}\left(0^{+}\right)$ via Eq. (4).

The indicator-restricted committor time-correlation function *C*_*AB*-*qq*_(*t*) is shown in Fig. 3 (top). It essentially mirrors the evolution of the committor time-correlation function *C*_*qq*_(*t*), rapidly converging toward the same slope. The reactive flux (bottom) shows that, like ${\dot{C}}_{qq}\left(t\right)$, $\dot{C}$_*AB*-*qq*_(*t*) reach a plateau within the lag-time *τ* of ∼0.08 ps (see the inset). In contrast, the activated-dynamics reactive flux ${\dot{C}}_{BB}\left(t\right)$ decays to reach its plateau value after a considerably longer time *τ*_m_ (∼12 ps in Fig. 3). The position time-correlation function ${\dot{C}}_{zz}\left(t\right)$ rises sharply and reaches its plateau faster than ${\dot{C}}_{BB}\left(t\right)$ but still more slowly than ${\dot{C}}_{qq}\left(t\right)$ and $\dot{C}$_*AB*-*qq*_(*t*). The behavior of *C*_*AB*-*qq*_(*t*) displays some very interesting similarity with the TPS study of van Erp *et al.*,^18^ who showed that the time-derivative of the population correlation function constructed from indicator functions that depends on the history of the trajectories (past and future) also reached the correct plateau value at very short time. Whereas a trajectory’s history (past and future) is explicitly visible in TPS,^16,17^ such information in TPT is incorporated through the committor probability function.^14^ Accordingly, it is the term $\left(q\left(\tau \right)-q\left(0\right)\right)$ in the TPT-based expression for *C*_*AB*-*qq*_(*t*) of Eq. (45), which carries information about a trajectory’s history and insures that only members of the transition paths ensemble are included, that dramatically affects the convergence of the reactive flux. While further analysis would be needed to formally relate the present analysis to TPS simulations,^18^ this highlights the importance of attaching some information about the reactive or non-reactive “fate” of a given trajectory to determine a transition rate, a concept that has been well understood, from the early days of transition state theory (TST)^2–4^ to more modern developments of the reactive flux formalism.^31^

The rapid convergence of the indicator-restricted committor time-correlation function *C*_*AB*-*qq*_(*t*) suggests that it may be possible to formulate an algorithm based on Eq. (45) that would rely on a local sampling like the activated-dynamics reactive flux formalism,^1,5,6^ but which would converge more rapidly toward the correct transition rate constant. This advantage, however, needs to be qualified. Although such an algorithm would converge within a short lag-time, an accurate estimate of the committor is required. The latter can be calculated for a given configuration by shooting trajectories to determine the probability of reaching state *B* before state *A*, although the most practical approach is to optimize a trial function *q*(**z**) via the variational principle based on Eqs. (23) and (24).^12,34,35^ The important question is whether the usage of an approximate committor, which introduces additional factors associated high order eigenvalues as shown by Eq. (32), can be utilized productively.

## V. CONCLUSION

The kinetic relaxation of a complex dynamical system dominated by two metastable states was examined from a Markov state eigenvalue spectral decomposition,^8–11^ a committor-based steady-state reactive flux,^7,13,14^ and the activated-dynamics reactive flux formalism.^1,5,6^ The present analysis was based on the assumption that the metastable states *A* and *B* were already known. Generally, discovering, identifying, and locating such metastable states in complex systems, however, can be a difficult challenge requiring specialized methods.^48–51^ Importantly, the robustness of the analysis and the overall shape of the committor function is typically not affected by the precise definition of the boundary states—this is one reason why the same definition was used for all three examples shown in Fig. 1 with very different free energy barriers.

The analysis showed that the different theoretical perspectives are consistent. This is most clearly displayed by the close correspondence between the eigenmode relaxation of Eq. (20), the steady-state reactive flux via Eq. (31), and the activated-dynamics formalism via Eq. (2). The spectral analysis of the effective propagator $P_{\tau }$ yields the eigenvalue $\lambda_{k}\left(\tau \right)=e^{-\mu_{k}\tau }$, with the second eigenvalue *μ*_2_ corresponding to the global relaxation time $\tau^{*}={\left(k_{AB}+k_{BA}\right)}^{-1}={\left(\mu_{2}\right)}^{-1}$ of the two-state system, while the net steady-state reactive flux from *A* to *B* is *J*_*AB*_ = *p*_*A*_ *k*_*AB*_.

The significance of the different time scales that appear in the analysis of the kinetic relaxation time of a two-state system was clarified. While Δ*t* is the microscopic time step used to generate the trajectory of the system, *τ* ≫ Δ*t* is the shortest lag-time that achieves Markovity for the effective propagator $P_{\tau }$ within the subspace **z**. The analysis also revealed the existence of an intermediate time scale *τ*_m_, which is associated with additional molecular processes that must decay to display the true global relaxation time of the two-state system. The intermediate time scale *τ*_m_ is larger than the lag-time *τ* but much smaller than the global relaxation time τ*. According to the spectral analysis, a good separation of time scales can be achieved if there is a large gap between the eigenvalues *μ*_2_ and *μ*_3_ such that $e^{-\mu_{3}\tau_{\text{m}}}\ll 1$ while $e^{-\mu_{2}\tau_{\text{m}}}\approx 1$. In particular, this intermediate time scale *τ*_m_ is displayed by the decay of the activated-dynamics reactive flux time-correlation function to a constant plateau corresponding to the transmission coefficient *κ*. However, it can also appear when an imperfect approximation for the committor is used in the expression for the steady-state reactive flux. In fact, there is a close correspondence between these two situations because the activated-dynamics reactive flux formalism is akin to using a Heaviside indicator function (population operator) to approximate the committor. This explains why the reactive flux with the indicator function is an effective route for a narrow sharp barrier but much less so in the case of a broad flat barrier. In this case, for example, the activated-dynamics reactive flux time-correlation function decays slowly to its plateau because the indicator function is affected by the diffusion process on top of the flat barrier.

An attractive feature of the activated-dynamics reactive flux formalism is the ability to calculate the forward transition rate constant from a local sampling of trajectories initiated at a given interface.^1,5,6^ While this provides an effective route to calculate the transition rate in the case of a narrow sharp barrier, it is much less effective in the case of a broad flat barrier. In contrast, the committor-based steady-state reactive flux rapidly converges to the correct value at the lag-time *τ* for both sharp and broad free energy barriers when the exact committor is used. The rapid convergence to the correct reactive flux within the time-lag *τ* and the absence of intermediate time scale *τ*_m_ is an attractive and robust feature of a committor-based steady-state reactive flux formulation. A tantalizing conjecture is whether this observation can serve to ascertain the Markovity of the effective propagator $P_{\tau }$ in the reduced subspace of the CVs.

It was shown that the most effective choice to define a one-dimensional reaction coordinate in the region of the transition state is along the gradient of the committor, ***∇****q*(**z**), in the multidimensional subspace of collective variables **z**. However, while the committor can be exploited as a useful reaction coordinate, simply treating the committor as a one-dimensional reaction coordinate in the activated-dynamics formalism does not alleviate the issues caused by slow relaxation and intermediate time scales. In that sense, the idea that the committor is the “perfect” reaction coordinate, as is sometimes suggested, needs to be interpreted carefully. It is possible to express the reactive flux as a local average via the indicator-restricted committor time-correlation function *C*_*AB*-*qq*_(*t*) in Eq. (45), which is one of the most attractive features of the activated-dynamics reactive flux formalism.^1,5,6^ Like Eq. (4), the forward transition rate constant can be calculated from a local sampling of very short trajectories initiated at a separating interface.

There is a large body of methods seeking to identify optimal reaction coordinates from complex dynamics,^11,33,47,52,53^ including dynamical self-consistency,^46^ memory reduction,^54^ multi-dimensional spectral gap optimization of order parameters (SGOOP),^55,56^ maximally predictive one-dimensional projection,^10^ and variational committor-based steady-state reactive flux.^12,34,35^ Also related are the dynamical Galerkin approximation (DGA) formulated to predict the long-timescale behavior from short-trajectory^34,35^ and nonparametric variational optimization of reaction coordinates.^57,58^ While challenging, the determination of an optimal reaction coordinate remains of great value, and a long term objective is to use these ideas to provide further guidance to theoretical frameworks built upon an optimized pathway.^29,59,60^

## Data Availability

The data that support the findings of this study are available within the article.

## APPENDIX A: DIFFUSIVE DYNAMICS AND COMMITTOR

If the dynamics is diffusive, we have^61^

$$\begin{array}{ll} J_{AB} & =-\int d\text{z}\int d{\text{z}}_{0}\;q\left(\text{z}\right)\;\left(\frac{d}{d\tau }{\left.P_{\tau }\left(\text{z}|{\text{z}}_{0}\right)\right|}_{\tau =0}\right)\;q\left({\text{z}}_{0}\right)\;\rho_{\text{eq}}\left({\text{z}}_{0}\right)\\ & =-\int d\text{z}\;q\left(\text{z}\right)\;\int d{\text{z}}_{0}\;\nabla \cdot \left[\text{D}\;\rho_{\text{eq}}\left(\text{z}\right)\nabla \left(\frac{\delta \left(\text{z}-{\text{z}}_{0}\right)}{\rho_{\text{eq}}\left(\text{z}\right)}\right)\right]\;q\left({\text{z}}_{0}\right)\;\rho_{\text{eq}}\left({\text{z}}_{0}\right)\\ & =-\int d\text{z}\;q\left(\text{z}\right)\;\nabla \cdot \left[\text{D}\;\rho_{\text{eq}}\left(\text{z}\right)\;\nabla \left(\int d{\text{z}}_{0}\;\frac{\delta \left(\text{z}-{\text{z}}_{0}\right)}{\rho_{\text{eq}}\left(\text{z}\right)}\;q\left({\text{z}}_{0}\right)\;\rho_{\text{eq}}\left({\text{z}}_{0}\right)\right)\right]\\ & =-\int d\text{z}\;q\left(\text{z}\right)\;\nabla \cdot \left[\text{D}\;\rho_{\text{eq}}\left(\text{z}\right)\nabla q\left(\text{z}\right)\right], \end{array}$$ (A1)

where the identity

$$\frac{d}{d\tau }{\left.P_{\tau }\left(\text{z}|{\text{z}}_{0}\right)\right|}_{\tau =0}=\nabla \cdot \left[\text{D}\;\rho_{\text{eq}}\left({\text{z}}_{0}\right)\nabla \left(\frac{\delta \left(\text{z}-{\text{z}}_{0}\right)}{\rho_{\text{eq}}\left({\text{z}}_{0}\right)}\right)\right]$$ (A2)

obtained from

$$\frac{d}{d\tau }P_{\tau }\left(\text{z}|{\text{z}}_{0}\right)=\nabla \cdot \left[\text{D}\rho_{\text{eq}}\left({\text{z}}_{0}\right)\nabla \left(\frac{P_{\tau }\left(\text{z}|{\text{z}}_{0}\right)}{\rho_{\text{eq}}\left({\text{z}}_{0}\right)}\right)\right]$$ (A3)

and $P_{0}\left(\text{z}|{\text{z}}_{0}\right)=\delta \left(\text{z}-{\text{z}}_{0}\right)$, has been used. It should be noted that the integralsare over the whole space rather than just the intermediate region to account for the discontinuity at the boundaries.

## APPENDIX B: MODEL COMMITTOR

The equilibrium population of the states *A* and *B* is given by

$$p_{B}\approx \int d\text{z}\;\rho_{\text{eq}}\left(\text{z}\right)\;q\left(\text{z}\right)$$ (B1)

and *p*_*B*_ = (1 − *p*_*A*_). When a very long trajectory is “colored” based on transitions to the two boundary regions, Eq. (B1) correctly provides the probability of the color ascribed to *B*.^7^ Using the model committor from Eq. (30), the backward propagation condition is^7,37^

$$\begin{array}{ll} \int d{\text{z}}^{\prime }\;q\left({\text{z}}^{\prime }\right)\;P_{\tau }\left({\text{z}}^{\prime }|\text{z}\right) & =-\left(\frac{a}{b-a}\right)\;\int d{\text{z}}^{\prime }\;\psi_{1}^{\text{L}}\left({\text{z}}^{\prime }\right)\;P_{\tau }\left({\text{z}}^{\prime }|\text{z}\right)\\ & \;+\left(\frac{1}{b-a}\right)\;\int d{\text{z}}^{\prime }\;\psi_{2}^{\text{L}}\left({\text{z}}^{\prime }\right)\;P_{\tau }\left({\text{z}}^{\prime }|\text{z}\right)\\ & =-\left(\frac{a}{b-a}\right)\;\psi_{1}^{\text{L}}\left(\text{z}\right)+\left(\frac{1}{b-a}\right)\;\lambda_{2}\;\psi_{2}^{\text{L}}\left(\text{z}\right)\\ & =q\left(\text{z}\right)+\left(\frac{\lambda_{2}-1}{b-a}\right)\;\psi_{2}^{\text{L}}\left(\text{z}\right)\\ & \approx q\left(\text{z}\right), \end{array}$$ (B2)

where |(*λ*_2_ − 1)/(*b* − *a*)| ≪ 1 (see below). We note that the vector $\psi_{1}^{\text{L}}\left(\text{z}\right)=1$ and the vector $\psi_{1}^{\text{R}}\left(\text{z}\right)=\rho_{\text{eq}}\left(\text{z}\right)$. From the construction,

$$\psi_{2}^{\text{L}}\left(\text{z}\right)=\left(b-a\right)\;q\left(\text{z}\right)+a\;\psi_{1}^{\text{L}}\left(\text{z}\right).$$ (B3)

The coefficients *a* and *b* are determined by the orthogonality and normalization conditions, $\left(\psi_{1}^{\text{L}}\cdot \psi_{2}^{\text{R}}\right)=0$ and $\left(\psi_{2}^{\text{L}}\cdot \psi_{2}^{\text{R}}\right)=1$. By virtue of orthogonality, we have

$$\begin{array}{ccc} 0 & = & \int d\text{z}\;\psi_{2}^{\text{L}}\left(\text{z}\right)\;\psi_{1}^{\text{R}}\left(\text{z}\right)\\ & = & \left(b-a\right)\int d\text{z}\;q\left(\text{z}\right)\;\psi_{1}^{\text{R}}\left(\text{z}\right)+a\int d\text{z}\;\psi_{1}^{\text{L}}\left(\text{z}\right)\;\psi_{1}^{\text{R}}\left(\text{z}\right)\\ & = & \left(b-a\right)\;p_{B}+a. \end{array}$$ (B4)

Hence, *a* = −(*b* − *a*) *p*_*B*_. By virtue of normalization, we have

$$\begin{array}{ccc} 1 & = & \int d\text{z}\;\psi_{2}^{\text{L}}\left(\text{z}\right)\;\psi_{2}^{\text{R}}\left(\text{z}\right)\\ & = & \int d\text{z}\;\psi_{2}^{\text{L}}\left(\text{z}\right)\;\rho_{\text{eq}}\left(\text{z}\right)\;\psi_{2}^{\text{L}}\left(\text{z}\right)\\ & = & \int d\text{z}\;\rho_{\text{eq}}\left(\text{z}\right){\left(\left(b-a\right)\;q\left(\text{z}\right)+a\;\psi_{1}^{\text{L}}\left(\text{z}\right)\right)}^{2}\\ & = & {\left(b-a\right)}^{2}〈q\;q〉+2\left(b-a\right)a〈q〉+a^{2}\\ & = & {\left(b-a\right)}^{2}\;\left(p_{B}-〈q\left(1-q\right)〉\right)+2\left(b-a\right)a\;p_{B}+a^{2}\\ & = & {\left(b-a\right)}^{2}\;\left(p_{A}\;p_{B}-〈q\left(1-q\right)〉\right). \end{array}$$ (B5)

This shows that $|\left(\lambda_{2}-1\right)/\left(b-a\right)|\approx \mu_{2}\tau \sqrt{p_{A}p_{B}}\ll 1$.

## APPENDIX C: REACTIVE FLUX FROM THE MODEL COMMITTOR

Using the model committor from Eq. (30) and the conditions established in Appendix B, the steady-state flux from *A* to *B* is

$$\begin{array}{ll} J_{AB} & =-\frac{1}{\tau }\left(\int d\text{z}\int d{\text{z}}^{\prime }\;q\left({\text{z}}^{\prime }\right)\;\rho_{\text{eq}}\left(\text{z}\right)\;P_{\tau }\left({\text{z}}^{\prime }|\text{z}\right)\;q\left(\text{z}\right)-\left\langle q\;q\right\rangle \right)\\ & =-\frac{1}{\tau }\left(\frac{a^{2}}{{\left(b-a\right)}^{2}}\int d\text{z}\int d{\text{z}}^{\prime }\;\psi_{1}^{\text{L}}\left({\text{z}}^{\prime }\right)\;P_{\tau }\left({\text{z}}^{\prime }|\text{z}\right)\;\psi_{1}^{\text{R}}\left(\text{z}\right)\right.\\ & \;\left.+\frac{1}{{\left(b-a\right)}^{2}}\int d\text{z}\int d{\text{z}}^{\prime }\;\psi_{2}^{\text{L}}\left({\text{z}}^{\prime }\right)\;P_{\tau }\left({\text{z}}^{\prime }|\text{z}\right)\;\psi_{2}^{\text{R}}\left(\text{z}\right)\right.\\ & \;\left.-p_{B}+\left\langle q\left(1-q\right)\right\rangle \right)\\ & =-\frac{1}{\tau }\left(p_{B}^{2}+\left(p_{A}\;p_{B}-\left\langle q\left(1-q\right)\right\rangle \right)\lambda_{2}-p_{B}+\left\langle q\left(1-q\right)\right\rangle \right)\\ & =\frac{1}{\tau }\;\left(p_{A}\;p_{B}\;\left(1-\lambda_{2}\right)-\left\langle q\left(1-q\right)\right\rangle \;\left(1-\lambda_{2}\right)\right). \end{array}$$ (C1)

Since ⟨*q*(1 − *q*)⟩ = ⟨*τ*_r_⟩ *J*_*AB*_, we have

$$J_{AB}=\frac{1}{\tau }\;p_{A}\;p_{B}\;\left(1-\lambda_{2}\right)\;{\left(1+\frac{\left\langle \tau_{\text{r}}\right\rangle }{\tau }\left(1-\lambda_{2}\right)\right)}^{-1}$$ (C2)

if *μ*_2_*τ* ≪ 1, leading to Eq. (31).
